# Recent Advances in Managing Spinal Intervertebral Discs Degeneration

**DOI:** 10.3390/ijms23126460

**Published:** 2022-06-09

**Authors:** Bogdan Costăchescu, Adelina-Gabriela Niculescu, Raluca Ioana Teleanu, Bogdan Florin Iliescu, Marius Rădulescu, Alexandru Mihai Grumezescu, Marius Gabriel Dabija

**Affiliations:** 1 “Gr. T. Popa” University of Medicine and Pharmacy, 700115 Iasi, Romania; costachescus@gmail.com (B.C.); bogdan.iliescu@gmail.com (B.F.I.); mariusdabija.md@gmail.com (M.G.D.); 2 “Prof. Dr. N. Oblu” Emergency Clinical Hospital, 700309 Iasi, Romania; 3 Department of Science and Engineering of Oxide Materials and Nanomaterials, Politehnica University of Bucharest, 011061 Bucharest, Romania; adelina.niculescu@upb.ro (A.-G.N.); grumezescu@yahoo.com (A.M.G.); 4 Department of Pediatric Neurology, “Dr. Victor Gomoiu” Children’s Hospital, 022102 Bucharest, Romania; raluca.teleanu@umfcd.ro; 5 “Carol Davila” University of Medicine and Pharmacy, 020021 Bucharest, Romania; 6 Department of Inorganic Chemistry, Physical Chemistry and Electrochemistry, University Politehnica of Bucharest, 011061 Bucharest, Romania; 7 Research Institute of the University of Bucharest—ICUB, University of Bucharest, 050657 Bucharest, Romania; 8 Academy of Romanian Scientists, Ilfov No. 3, 050044 Bucharest, Romania

**Keywords:** intervertebral disc degeneration, regeneration strategies, intervertebral disc repair, cell-based therapies, artificial intervertebral discs

## Abstract

Low back pain (LBP) represents a frequent and debilitating condition affecting a large part of the global population and posing a worldwide health and economic burden. The major cause of LBP is intervertebral disc degeneration (IDD), a complex disease that can further aggravate and give rise to severe spine problems. As most of the current treatments for IDD either only alleviate the associated symptoms or expose patients to the risk of intraoperative and postoperative complications, there is a pressing need to develop better therapeutic strategies. In this respect, the present paper first describes the pathogenesis and etiology of IDD to set the framework for what has to be combated to restore the normal state of intervertebral discs (IVDs), then further elaborates on the recent advances in managing IDD. Specifically, there are reviewed bioactive compounds and growth factors that have shown promising potential against underlying factors of IDD, cell-based therapies for IVD regeneration, biomimetic artificial IVDs, and several other emerging IDD therapeutic options (e.g., exosomes, RNA approaches, and artificial intelligence).

## 1. Introduction

**Figure 1:**
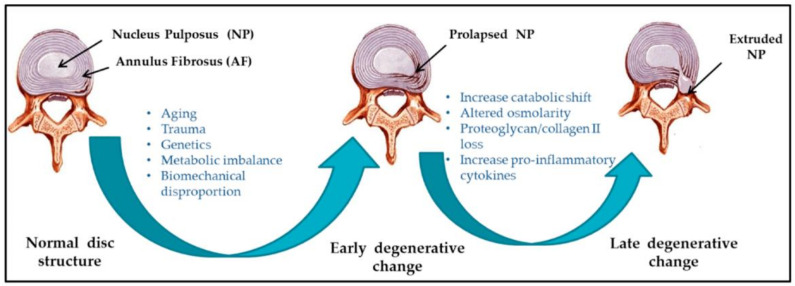
Schematic representation of intervertebral disc (IVD) pathophysiology during degeneration. Reprinted from an open access source [6].

Spinal disorders cover many pathologies (e.g., osteoporotic fractures, disc degeneration, spondylolisthesis, tumors, and large deformations), being among the most prevalent and costly medical conditions in Western societies [1]. Particularly, the degeneration of intervertebral discs (IVDs) has been considered the major cause of low back pain [2,3,4,5]. Mostly correlated with age, but not exclusively, IVD degeneration (IDD) can further aggravate into connected diseases, such as disc herniation, spinal canal stenosis, and spinal deformities [3,6,7]. Thus, proper and prompt treatment should be given to IDD patients to avoid the condition worsening (Figure 1).

Current IDD management options include surgical treatments, such as discectomy, arthroplasty, and spinal fusion, or medications to alleviate associated pain. Nonetheless, therapeutic agents, including analgesics and anti-inflammatory drugs, represent only symptomatic treatments that merely delay or mask IDD, whereas surgical methods are used as a last resort because they are associated with a high risk of intraoperative and postoperative complications, limit spine mobility, and fail to maintain the function of the treated IVD in the long term. Moreover, surgical interventions may alter the mechanical strain fields in the IVD after removing disc material, affecting the biomechanics of spinal segments. For instance, after surgical treatments, such as fusion or total disc replacement, the load and motion are transmitted by the rigid connection and the articulating contact. In contrast, in natural IVDs, they are provided by viscoelasticity, thanks to the deformable nature of this soft tissue. Therefore, more direct and effective treatments that address the fundamental underlying pathological conditions must be sought and implemented in practice [7,8,9,10,11].

In this context, there is an increasing research interest in finding new therapeutic agents that would not only alleviate pain but would also help regenerate damaged IVDs through the restoration of tissue homeostasis [12]. Recent advances in regenerative medicine and tissue engineering also hold promise for developing better-performing IDD treatment options capable of inhibiting or reversing degenerative processes [7,13,14].

Therefore, this paper aims to set the background concerning the pathogenesis and etiology of IDD, further focusing on novel strategies for managing this disease. Specifically, this review presents the bioactive compounds that have been reported as beneficial for repairing IVDs, the newly developed cell-based therapies for IVD regeneration, the artificial IVDs that can replace their damaged native counterparts, and several other emerging strategies, including the use of exosomes, RNA approaches, and artificial intelligence. In this respect, this work reviews English language papers in the field, published between 2012 and 2022 (predominantly from the last 7 years), retrieved from the Science Direct and Google Scholar databases.

## 2. Pathogenesis and Etiology of Intervertebral Disc Degeneration

Human spine IVDs have a unique structure that endows them with the necessary flexibility and mechanical support for allowing the movement of the spinal column and dissipating applied loads evenly over the vertebral bodies as the spine is flexed or extended [15]. Anatomically, the IVD is a large and avascular fibrocartilaginous tissue located between every two neighboring vertebrae. It consists of three main parts: the central nucleus pulpous (NP), surrounding collagenous annulus fibrous (AF), and the two cartilaginous endplates (CEP) that separate AF and NP from the vertebral bodies [16,17,18,19,20]. NP is the hydrophilic core of IVD, composed of collagen II and elastin fibers embedded in an aggrecan-containing gel. Through their fixed charge density, aggrecan molecules generate osmotic pressure, thus, contributing to the highly hydrated nature of the NP, preserving IVD height, and distributing the loading across the CEP. NP cells exhibit a low density; however, they are considered notochordal in origin before becoming chondrocyte-like with aging. The NP is laterally enclosed by AF, which comprises 15 to 25 lamellae (concentric rings). There are parallel collagen fibers within each lamella and perpendicular collagen fibers between the adjacent lamellae, endowing the structure with tensile strength. AF’s primary functions are to contain the NP and maintain its pressurization under compressive loads. The CEP represents a thin horizontal hyaline cartilage layer through which the IVD connects to each vertebra. Moreover, it forms the main source of nutrient, waste, and gas exchange for the avascular discs [18,19].

The optimal physiological status of IVDs in humans is achieved in their thirties. Given that IVDs are avascular and lack a strong regenerative capacity, these structures are prone to degeneration. Intervertebral disc degeneration (IDD) is a multifactorial process that can result from various causes, counting aging, injury, wrong posture, abnormal loading, and environmental and genetic factors [20,21]. The disappearance of large vacuolated notochordal cells in NP around the age of 10 and the calcification of the CEPs are two processes that initiate and exacerbate IDD [17]. The degenerative cascade is frequently triggered by an imbalance between catabolic and anabolic processes in the IVDs [22]. This disequilibrium may be associated with CEP damage that alters the NP’s mechanical loading, stimulating IVD metabolic disturbances. In addition, inadequate nutrition lowers IVD’s capacity to respond to increased load or injury, further propagating the degenerative cycle [12,18].

Disturbed permeability and nutrient transport resulting from CEP calcification also lead to the creation of a hypoxic and acidic medium that further impairs IVD cells’ normal activity of synthesizing and supporting the extracellular matrix (ECM) [12]. Subsequent to ECM degradation, tissue strength weakening, neoinnervation, and neovascularization occur [18,22]. These processes ultimately lead to disc bulging, loss of NP and water content, and subsequent disc height loss [22]. IDD structural changes lead to abnormal stress distribution of the spine, the subsequent biomechanical compensation resulting in a variety of potential complications, such as intervertebral space stenosis, intervertebral foramina stenosis, nerve entrapment and irritation, chronic low back pain, herniation, osteophytosis, spondylolisthesis, and spondylosis [21,22,23].

**Figure 2:**
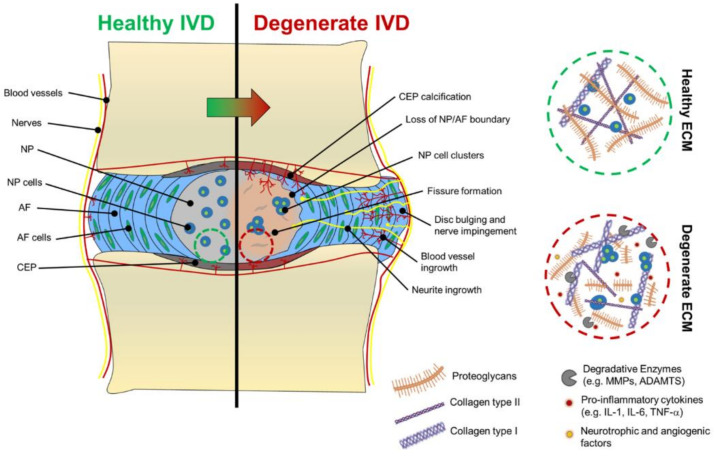
Visual summary of the macroscopic and microscopic changes during intervertebral disc degeneration (IDD). Reprinted from an open access source [24].

For better clarity, the macroscopic and microscopic changes in the IVD during degeneration are schematically represented in Figure 2.

From a mechanistic point of view, IDD is associated with a series of underlying factors, including increased levels of reactive oxygen species (ROS), insufficient autophagic flux, increased levels of matrix metalloproteinases (MMPs) in the IVD, activation of the senescent signal pathways to induce cell cycle arrest of NP cells and apoptosis [18,23]. Another important factor in IDD development is inflammation, as proinflammatory cytokine and chemokine production has been associated with IDD and LBP. Specifically, overproduction of interferon-gamma (IFN-γ), tumor necrosis factor-alpha (TNF-α), and interleukins (IL-1, 2, 6, 8, and 17) by the inflammatory cells from IVDs can start the cascade of tissue degeneration. In more detail, IL-1β, IL-6, IL-8, and TNF-α have been recognized as essential contributors to the development of neuropathic pain through irritation of ingrowing nerves. IL-17 was observed to promote ECM degradation, enhance inflammatory response, induce neoangiogenesis, and inhibit NP cell autophagy and proliferation. IFN-γ was reported to affect tissue-specific macrophages in NP. Furthermore, several angiogenic and neurogenic factors have also been linked to the degeneration of IVDs, conducting to blood vessels and nerve in-growth [12,20,25,26,27,28].

**Figure 3:**
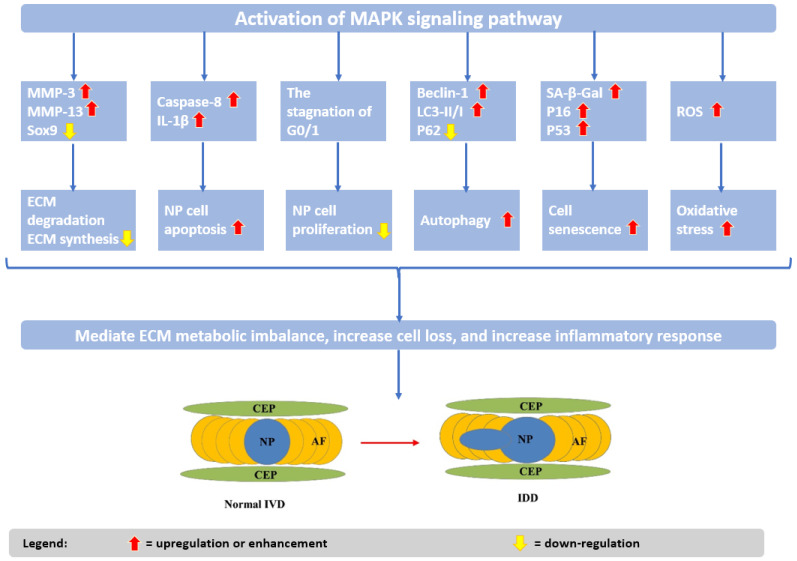
Effects of mitogen-activated protein kinase/extracellular signal-regulated kinase (MAPK/ERK) signal transduction on IDD. Adapted from an open access source [29].

In the above-described processes, the mitogen-activated protein kinase (MAPK)/extracellular signal-regulated kinase (ERK) signaling pathway was noticed to play an important role (Figure 3). Thus, understanding its implications in IVD signal transduction and targeting this pathway may lead to novel options for preventing or reversing IDD [29].

Recent investigations of IDD etiology revealed new insights into disc cell phenotypes and potential biomarkers. Specifically, Cherif et al. [30] proposed a list of specific cell-type biomarkers (i.e., *C2orf40*, *MGP*, *MSMP*, *CD44*, *EIF1*, *LGALS1*, *RGCC*, *EPYC*, *HILPDA*, *ACAN*, *MT1F*, *CHI3L1*, *ID1*, *ID3,* and *TMED2*) and a list of predictive IDD genes (i.e., *MT1G*, *SPP1*, *HMGA1*, *FN1*, *FBXO2*, *SPARC*, *VIM*, *CTGF*, *MGST1*, *TAF1D*, *CAPS*, *SPTSSB*, *S100A1*, *CHI3L2*, *PLA2G2A*, *TNRSF11B*, *FGFBP2*, *MGP*, *SLPI*, *DCN*, *MT-ND2*, *MTCYB*, *ADIRF*, *FRZB*, *CLEC3A*, *UPP1*, *S100A2*, *PRG4*, *COL2A1*, *SOD2*, and *MT2A*). These advances hold great promise for improving diagnostic and therapeutic possibilities.

## 3. Strategies for Managing Intervertebral Disc Degeneration

Given the complexity of IDD etiology and pathophysiology and the lack of satisfactory treatment, there is a pressing need to develop new strategies for dealing with this disease. Recent attention has been drawn by regenerative medicine approaches that aim to restore living tissues with the help of various scaffolds, cells, growth factors, and biologically active compounds. In this regard, the following subsections review the novelties in the field.

### 3.1. Key Compounds in Intervertebral Disc Repair

Pain reliever medication is given to patients with symptomatic degenerative disc disease, but it only works in the short term, decreasing inflammation and providing temporary anesthesia in the area where the irritated nerve is involved [17]. To achieve longer-term results and better outcomes, the focus must be directed to the factors driving the pathological alterations. Thus, drug development should take into account and overcome issues, such as excessive apoptosis of NP cells, the imbalance between anabolism and catabolism of ECM, rupture of AF, and degeneration and calcification of CEP [23].

In this respect, promising evidence has been obtained from recent studies concerning IDD treatment through the administration of several bioactive compounds and growth factors, as is further elaborated.

#### 3.1.1. Melatonin

Increasing evidence supports the benefits of melatonin administration for alleviating IDD produced by fibro-puncture, inflammation, or oxidative stress stimulation. Melatonin was seen to maintain the structural integrity and orderliness of the IVD by facilitating cell survivability in the CEP, AF, or NP, stimulating matrix anabolism, inhibiting vascular invasion, mitigating calcification, and enhancing the speed of damage repair [23,31,32,33].

Several recent studies investigated the roles of melatonin more deeply, searching for its mechanisms of action against IDD. For instance, Zhang et al. [34] revealed that melatonin could modulate ECM remodeling induced by IL-1β by increasing collagen II and aggrecan expression levels and decreasing MMP-3 levels in rat tail puncture models. Through these mechanisms, melatonin can aid in the restoration processes of IVD, being a potential therapeutic agent against IDD. Similar findings were also reported by Chen and colleagues [35], who discovered that melatonin disrupts the IL-1β/NF-κB-NLRP3 inflammasome activation positive feedback loop in vitro and in vivo. In more detail, melatonin administration decreased NLR family pyrin domain containing 3 (NLRP3), p20, and IL-1β levels by inhibiting nuclear factor kappa B (NF-κB) signaling and downregulating mtROS production in human NP cells treated with IL-1β and a rat needle puncture IDD model. Complementarily, Ge et al. [36] reported that melatonin treatment produced the upregulation of collagen II and aggrecan while downregulating collagen X. In addition, this compound was noted to significantly enhance the activity of the ERK signaling pathway, affecting the biological properties of IDD, both in vivo (i.e., a rabbit model of IDD) and in vitro (i.e., human NP cells). Moreover, according to Wu et al. [37], melatonin treatment alleviates osteochondral destruction as fewer receptor activators for nuclear factor-κB ligand (RANKL) and tartrate-resistant acid phosphatase (TRAP)-stained positive cells were present in the cartilaginous endplates of treated rats.

#### 3.1.2. Estrogen

Estrogen is known to play an important role in various biological processes, participating in the etiology and pathogenesis of many diseases. In particular, estrogen deficiency is a contributing factor in the onset of IDD in postmenopausal women [20,38,39]. Hence, estrogen administration is expected to have the opposite effect, reverting or at least retarding the progression of IDD.

Research studies revealed estrogen could effectively alleviate IDD development by inhibiting IVD cells’ apoptosis through the suppression of inflammatory cytokines IL-1β and TNF-α. Moreover, it reduces catabolism through MMP inhibition, upregulates integrin α2β1 and IVD anabolism, activates the phosphatidylinositol-3-kinase/protein kinase B (PI3K/Akt) pathway, decreases oxidative-stress-induced damage, and promotes autophagy [40].

In a study by Liu et al. [41], it was reported that 17β-Estradiol (E2) could protect against rat IDD by diminishing the expression of caspase-3 and intracellular MMPs, including MMP-3 and MMP-13, and upregulating collagen Type II. Additionally, Wang et al. [38] suggested that E2 could inhibit NP cell apoptosis by suppressing the NF-κB signal pathway, being a promising mechanism for preventing the occurrence or progression of IDD, both in vitro (i.e., human NP cells collected from women) and in vivo (i.e., rat coccygeal IDD model).

Nonetheless, estrogen-related complications (e.g., hypertension, cerebrovascular accident, myocardial infarction, venous thromboembolism, pulmonary embolism, exacerbation of epilepsy, and endocrine-related cancers) should not be overlooked when considering estrogen replacement therapy for retarding IDD [20,40,42,43]. In this context, some other chemicals that can replace estrogen started to gain research interest [20], including selective estrogen receptor modulators (SERM) [44,45] and phytoestrogens [46,47].

#### 3.1.3. Naringin

Naringin is a natural compound with many pharmacological effects that has also attracted attention for IDD treatment [48]. Li and colleagues [49] reported that this flavonoid could effectively promote the proliferation of NP cells in vitro, improving the recuperation of the cells from degeneration through the increase in aggrecan, bone morphogenetic protein 2 (BMP-2), and SRY-box transcription factor 6 (Sox6), and decrease in TNF-α and MMP3 expression.

In addition, a study conducted by Zhang et al. [50] revealed that naringin could protect NP cells against apoptosis and alleviate IDD in vivo (i.e., puncture-induced rat model) through an autophagy-regulation-related mechanism. Specifically, it was observed that naringin reduced the occurrence of oxidative-stress-induced apoptosis in NP cells and increased the expression of autophagy markers light chain 3-II/I (LC3-II/I) and beclin-1. Research findings also indicated that autophagy regulation of naringin might be related to AMP-activated protein kinase (AMPK) signaling, and administering this treatment may also modulate the expression of collagen II, aggrecan, and MMP 13 to support ECM. Moreover, according to Nan et al. [51], the activation of the ROS-mediated PI3K/Akt pathway may be a potential mechanism for naringin’s ability to attenuate H_2_O_2_-induced NP cells apoptosis and mitochondrial dysfunction, as indicated by their results obtained from in-vitro tests on rat-NP-derived mesenchymal stem cells (MSCs). Another in-vitro study, conducted by Devraj et al. [52], also suggested that naringin can bind with genes identified as potent inflammation inhibitors, thus, serving as anti-inflammatory agents in treating low back pain and sciatica.

#### 3.1.4. Icariin

Icariin is another compound that showed promising results in preventing H_2_O_2_-induced NP cell apoptosis. In-vitro and in-vivo studies on rat models [53,54] indicated that icariin treatment significantly decreased intracellular ROS levels, downregulated mitochondrial membrane potential, reduced the expression of Caspase-3 and BCL-2-associated X protein (Bax), and upregulated phosphorylated Akt (p-Akt) and B-cell lymphoma 2 (BCL-2). Hua et al. [54] additionally reported that icariin can ameliorate IDD by promoting nuclear factor erythroid 2-related factor 2 (Nrf-2) activity and preserving ECM in human NP cells. The same research group [55] had previously proved that this natural compound is useful in IDD treatment due to its anti-inflammatory effect. Particularly, icariin was observed to suppress IL-1β-induced activation of MAPK- and NF-ΚB-related signaling pathways, exerting a protective effect on human NP cells.

Alternatively, Zhang et al. [56] demonstrated the ability of icariin to recruit stem cell niches (SCNs) within the intervertebral disc region (ISN)-derived stem cells (ISN-SCs). The authors showed that icariin treatment promoted the migration of stem cells in IDD by upregulating chemotactic cytokines, such as insulin-like growth factor 1 (IGF-1), transforming growth factor-beta (TGF-β), stromal cell-derived factor-1 (SDF-1), and C-C motif chemokine ligand 5 (CCL-5).

#### 3.1.5. Resveratrol

Promising outcomes also arise from using resveratrol for ameliorating IDD, especially due to its antioxidant properties, manifested through regulating mitochondrial dysfunction or ROS elimination [57,58,59,60,61]. Wang et al. [62] reported that resveratrol can inhibit ROS generation and enhance the activity of the PI3K/Akt pathway, demonstrating protective effects against high glucose-induced rat NP cell apoptosis and senescence. In agreement with these results are the findings of Gao and colleagues [63], who described the protective role of resveratrol in enhancing NP matrix synthesis under oxidative damage. Moreover, the authors observed that resveratrol treatment considerably upregulated the expression of aggrecan and collagen, promoted glycosaminoglycan (GAG) production, and increased the expression of autophagy-related markers (i.e., Beclin-1 and LC-3) in rat NP cells. Similarly, through in-vitro tests on human NP cells, Liu et al. [64] noticed the ability of resveratrol to rescue TNF-α-induced downregulation of collagen II and aggrecan production, indicating the potential of this polyphenol compound in treating IDD.

Furthermore, Jiang et al. [65] successfully investigated the anti-inflammatory potential of resveratrol via in-vitro tests on rat NP cells. The researchers reported that resveratrol could partly suppress IL-1β-induced NP cell apoptosis, attenuating inflammation-response-induced disc degeneration. Interesting results have also been obtained with the combined treatment of resveratrol and E2. According to the research conducted by Bai et al. [66] on human NP cells, the synergic therapy effectively inhibited IL-1β-induced NP cell apoptosis via the PI3K/AKT pathway, recovering cell viability. In more detail, this combined treatment increased the activated phospho-mammalian target of rapamycin (P-mTOR) and phospho-glycogen synthase kinase-3 beta (P-GSK-3β), leading to the downregulation of Caspase-3.

#### 3.1.6. Quercetin

Quercetin, with its recognized antioxidant and anti-inflammatory properties, has been rendered beneficial in treating degenerative diseases, including IDD. Nonetheless, its mechanisms of action remain unclear [67,68,69,70,71]. In this respect, several recent studies focused on elucidating the way in which quercetin can prevent IDD.

Wang et al. [72] noted that this flavonoid could inhibit the apoptosis of NP cells and ECM degeneration induced by oxidative stress via the sirtuin 1 (SIRT1)-autophagy pathway in vitro, while in-vivo studies demonstrated that quercetin could alleviate IDD progression in rats. Zhang and colleagues [67] also observed the relation between quercetin and autophagy; yet another mechanism was proposed. The authors reported that this compound partially inhibited the p38 MAPK signaling pathway, leading to autophagy, indicating that quercetin can protect NPCs against apoptosis and prevent ECM degeneration by modulating the p38 MAPK-autophagy pathway. These effects were first observed in vitro on tert-butyl hydroperoxide-treated NP cells and were further confirmed in vivo by a rat-tail-puncture-induced IDD model. Alternatively, Shao et al. [73] revealed that quercetin inhibited IL-1β-induced activation of the NF-κB pathway cascades, suppressing SASP factor expression and senescence phenotypes in NP cells, both in vitro and in vivo. Hence, they concluded that quercetin is a senolytic agent with significant potential in IDD therapy.

#### 3.1.7. Berberine

Berberine is an alkaloid compound remarked for its useful properties, counting antimicrobial, anti-inflammatory, antioxidative, and anti-apoptotic effects [74,75,76]. Tackling these advantageous activities, several studies have investigated berberine as a potential therapeutic agent in IDD.

Luo et al. [77] explored the ability of berberine to prevent NP cell apoptosis under oxidative damage, attempting to unravel its potential underlying mechanisms. Their in-vitro (i.e., human NP cells) and in-vivo (i.e., IDD rat model) studies revealed that this small molecule prevents apoptosis by modulating endoplasmic reticulum stress and autophagy, being a promising pharmacological candidate for treating IDD. Chen et al. [74] also noticed the role of berberine in stimulating autophagy as a protective mechanism against NP cell apoptosis and ECM degeneration. Moreover, the authors performed in-vivo studies that showed an increased expression of LC3 in disc cells after berberine treatment, preventing the occurrence of IDD in a needle-puncture-induced rat model.

In addition, a study conducted by Lu and colleagues [78] demonstrated that berberine could aid in IDD therapy through the inhibition of the inflammatory response. More specifically, this compound attenuated the upregulation of ECM-catabolic factors and the downregulation of ECM-anabolic factors and protected human NP cells from IL-1β-induced apoptosis.

#### 3.1.8. Metformin

Metformin was also suggested to have beneficial activity in IDD treatment. This compound was noted to stimulate autophagy, reduce local mechanical hyperalgesia, and inhibit inflammatory responses in NP and AF cells, protecting against apoptosis and senescence [79,80]. An in-depth study performed by Chen et al. [81] concerning its mechanisms of action revealed that metformin activates autophagy in NP cells in a dose- and time-dependent manner, promotes the expression of anabolic genes, such as *Col2a1* and *Acan*, and inhibits the expression of catabolic genes, such as *MMP3* and *ADAMTS5*. Han et al. [82] also concluded that metformin could be efficient against IDD due to its ability to enhance anabolism while inhibiting catabolic production and cell senescence in rabbit AF stem cells. Moreover, the authors also reported anti-inflammatory effects for metformin via blocking the HMGB1 translocation.

More recently, Hu and colleagues [83] reported that metformin could reduce hypermethylation of the SOX9 promoter, restoring the expression of SOX9 in IDD cells and rabbit models. Further, the increase in SOX9 levels stimulated the expression of miR-202-3p, inhibiting MMP-1 expression. Additionally, Ramanathan et al. [84] suggested lately that metformin acts at the gene transcription level via the NF-κB pathway, reducing inflammation and improving matrix production in rat AF cells.

#### 3.1.9. Vitamin D

Vitamin D is recognized for its immunomodulatory properties, involvement in osteocartilaginous metabolism, and potential role in IVD pathophysiology [85]. Specifically, vitamin D and vitamin D receptors have been noted to be employed in different autophagy steps [86]. Moreover, a deficiency in vitamin D (i.e., serum concentration < 10 ng/mL) has been linked with lumbar disc degeneration and low back pain in postmenopausal women [87]. Thus, it can be expected that vitamin D administration would have beneficial effects on IDD patients.

In this respect, Huang et al. [88] investigated the potential of vitamin D as IDD treatment in mice models. The authors reported dose-dependent retardation of the disease, observing a reduction in inflammatory responses, oxidative stress, apoptosis, and cell aging. In comparison with control groups, mice treated with vitamin D also exhibited increased collagen II levels and decreased collagen X levels. The underlying mechanism of IDD retardation was considered the inhibition of the NF-κB pathway. According to De Luca et al. [85], vitamin D also promotes the upregulation of aggrecan in inflammatory conditions.

For clarity, the above-discussed compounds are summarized in Table 1, offering an at-glance perspective on their relevant effects on IDD and mechanisms of action.

#### 3.1.10. Growth Factors

As it has been observed that reduced growth factor supply at the level of IVD increases the rate of disc cell senescence, the administration of growth factors began to be considered a potential therapeutic strategy. Injecting various growth factors (e.g., members of the transforming growth factor-β (TGF-β) superfamily, IGF-1, growth differentiation factor 5 (GDF-5), BMP-2, BMP-7, and platelet-derived growth factor (PDGF)) into the IVD can stimulate ECM synthesis, prevent cell senescence induced by oxidative stress, relieve inflammation, restore the balance of anabolic and catabolic activities, and delay the degeneration process [13,17,18,89]. In more detail, TGF-β1 and TGF-β3 can induce discogenic differentiation, direct cells towards a fibrocartilaginous phenotype, and reduce the damage caused by degeneration. IGF-1 levels are downregulated in IDD, while its administration to degenerated discs has been shown to prevent disc cell senescence induced by oxidative damage. GDF-5 is of particular interest to IDD treatment due to its influence on joint and skeletal development via matrix production and disc cell proliferation. BMPs are recognized for their role in bone homeostasis and cell differentiation into bone and cartilage. In particular, BMP-7 and BMP-2 treatment can aid in proteoglycan metabolism and ECM synthesis in IVD cells exposed to inflammatory factors. Moreover, PDGF is involved in the angiogenesis and growth of existing blood vessels, also being able to retard cellular breakdown and turnover through its anti-catabolic activity [13,18].

Nonetheless, the results obtained through in-vitro and in-vivo studies must be validated in clinical trials before applying growth-factor-based strategies in clinical practice [13]. In this respect, several clinical trials have been reported in the field (Table 2) that might soon offer promising outcomes and bring advances in managing IDD.

### 3.2. Cell-Based Strategies

Various cell-based therapies, counting the delivery of autologous, allogeneic, or xenogeneic sources of primary or stem cells, with or without encapsulation in a biomaterial, have attracted tremendous scientific interest in tissue regeneration. Cells can be injected intravenously, transplanted at the desired site within a scaffold, or recruited from a patient’s own tissue in order to take advantage of the self-repair processes [24].

The minimally invasive transplantation of different cells (e.g., MSCs, induced pluripotent stem cells (iPSCs), embryonic stem cells (ESCs), NP-derived cells, chondrocyte-like cells) has the potential to support the IVD by differentiating into IVD-like cells and/or by secreting trophic and anti-inflammatory factors. Thus, cell-based therapies can be used to regenerate or at least slow down degeneration, whereas combining cells with a biocompatible material can additionally provide initial mechanical stability and protection after implantation [3,7,90].

MSCs are the most clinically evaluated cell type for disc regeneration therapy, being recognized for their ability to self-renew and differentiate into many tissue types, including chondrogenic and IVD-cell lineages [7,90]. MSCs have also been noted to preserve their multi-directional differential potential after continuous subculture and cryopreservation, being ideal cells for repairing damage caused by senescence and pathological changes [9]. Other favorable characteristics of MSCs include their ease of isolation, engraftment capacity, safety profile, and immunomodulatory properties. Moreover, MSC treatments are considered safe and well-tolerated, potentially limiting the inflammatory environment of the IVD upon transplantation and leading to no severe adverse effects. However, occasional mild-pain-related adverse events might occur [2,19].

Given the plethora of advantages presented by MSCs, researchers actively investigated various strategies to use these cells for repairing degenerated IVDs. For instance, Dong et al. [91] developed flower-stacked blended polymeric porous microspheres as carriers of connective tissue growth factor signaling molecules and bone-marrow-derived MSCs. The designed constructs displayed excellent cell compatibility and adhesive ability, provided effective in-vitro chondrogenic differentiation of stem cells, and boosted GAG production, being a promising IVD therapy.

Another combined bioactive delivery matrix for degenerated IVD was proposed by Kim and colleagues [92], who prepared leaf-stack porous particles loaded with TGF-β3 and human-bone-marrow-derived MSCs. These particles created a suitable medium for chondrogenic differentiation of the carried stem cells, effectively inducing IVD regeneration in a beagle dog model. Therefore, the authors concluded this strategy to be a potential candidate system for bioactive delivery in IDD therapy.

Alternatively, other research groups investigated the therapeutic efficacy of stem cell incorporation into gels. For example, Ukeba et al. [93] used a combination of ultra-purified MSCs (i.e., rapidly expanding clones, RECs) and a bioresorbable and good manufacturing practice (GMP)-compliant in-situ-forming ultra-purified alginate gel for IVD regeneration after discectomy in a sheep model of severe IVD degeneration. This hybrid strategy led to a significant increase in the gene expressions of NP cell markers, growth factors, and ECM, enhancing IVD regeneration. Thus, the developed method has promising translational potential for treating herniations in degenerative human IVDs.

An interesting possibility is also offered by Jia et al. [94], who recently fabricated an injectable glycerol crosslinked polyvinyl alcohol gel cultured with NP cells. The gel mimicked the mechanical properties of NP, exhibiting viscoelastic property and withstanding cyclic deformation with an effective energy-dissipating capability. In vitro, it was noted to considerably downregulate the expression of catabolic markers, preserve anabolic markers levels, maintain cell viability and proliferation, and lower apoptosis rate in NP cells. Moreover, in-vivo studies demonstrated the gel’s ability to ensure NP structural integrity, IVD height, and relative water content in IDD models, stimulating fibrous repair.

Recent focus has also been directed towards using bone marrow aspirate concentrate (BMAC) as a therapeutic option for IDD, as it contains intrinsic stem cells and growth factors. Wolff et al. [95] reported that intradiscal BMAC administration effectively reduced pain and improved function, especially in patients with relatively higher initial pain. Another study, by El-Kadiry and colleagues [96], demonstrated the potential of BMAC in reducing LBP, ameliorating mobility, and inducing parallel anatomical disc changes in degenerative joint disease to a greater extent in IDD compared to facetogenic pain subgroups. Thus, the authors concluded that BMAC might further be used as a substitute for IDD surgery in following wider-scale studies.

### 3.3. Artificial Intervertebral Discs

In recent years, scientific efforts have been directed toward designing artificial IVDs that can mimic the kinematics and dynamics of natural discs. A variety of materials, including metals, ceramics, polymers, and composites, have been explored to create suitable devices for managing IDD. Nonetheless, artificial IVD development faces very slow progress in comparison to other artificial joint technologies, especially due to the complex physiology and mechanics of IVDs. Moreover, the currently available devices present disadvantages, such as wear and mismatch between the implant and the natural tissues [97]. Particularly, the majority of implants used for total disc replacement (TDR) surgery are designed as a ball and socket pair, a structure that wears the implant, limiting its life [98]. Furthermore, metal-on-metal TDR devices contain structures with many interfaces (e.g., endplate/bone interface, endplate screws and bone, articulating surfaces) that may produce debris and/or ions and potentially generate adverse biological responses that lead to device failure and revision [99]. Other concerns regarding the exposure of tissues to metallic wear debris and ions count their potential to induce inflammation, genotoxicity, cytotoxicity, hypersensitivity and pseudotumor formation, and the ability to compromise the barrier function of the outer meningeal layer [100].

Thus, research focused on using more tissue-friendly materials or biodegradable implants to reduce the risk of long-term complications [1]. Biocompatible materials that retain the native biomechanics and promote tissue integration, such as in-situ hydrating synthetic polymers (e.g., copolymeric hydrogel encased in a polyethylene fiber jacket polyacrylonitrile and polyacrylamide (PDN™)) and in-situ forming synthetic polymers (e.g., chemically crosslinked biomaterial NuCore™, BioDisc™), are appealing for NP replacement. The main advantage of these synthetic polymers is the possibility of controlling their degree of swelling. Nonetheless, disadvantages, including excessive implant stiffness, endplate overloading and fracture, and fragmentation of gel upon swelling, should not be overlooked [6].

Poly(2-hydroxyethylmethacrylate) (PHEMA) hydrogels have also been considered for designing innovative artificial IVDs. They present beneficial properties, such as biocompatibility, high permeability, and high hydrophilicity, but they are limited by insufficient mechanical strength in the swollen state. To achieve better mechanical performance, hydrophobic components (e.g., poly(caprolactone) (PCL) and polymeric fibers) can be incorporated into these hydrogels. The addition of hydroxyapatite and/or calcium phosphate is a suitable alternative for making endplates, given that these bioactive materials can stiffen polymers [97].

A variety of other hydrogel-based artificial IVDs have been recently proposed. For instance, Guo et al. [101] designed a silk fibroin/nano-hydroxyapatite hydrogel mixed with icariin. The as-described hydrogel could promote the proliferation and differentiation of bone marrow MSCs into NP-like cells, increase cell–matrix synthesis, and promote the repair of degenerative IVD.

Another example is offered by Wu et al. [102], who fabricated a 3D biodegradable scaffold made of polylactic acid (PLA) and a double-network hydrogel. In-vitro studies proved the biocompatibility of the designed scaffold and the good viability of bone marrow MSCs in the hydrogel. Moreover, in vivo, the cell-laden IVD scaffold could preserve the disc space and produce a new ECM.

Alternatively, Du and colleagues [103] engineered a biomimetic AF-NP composite with circumferentially oriented PCL microfibers seeded with AF cells, with an alginate hydrogel encapsulating NP cells as a core. The designed artificial IVDs exhibited progressive tissue formation over time, as indicated by the deposition and organization of ECM and enhanced mechanical properties. Due to their similarity in form and function with native discs, these composites are promising candidates for IVD replacement.

To mimic the structures and biomechanics of AF and NP, Yang et al. [11] proposed the use of a polyvinyl alcohol (PVA)-bacterial cellulose (BC) composite. Their artificial IVD consists of a PVA-based gelatinous core carrying negative charges and BC outer multilayers that hold the nucleus together and provide tensile strength. The composite displayed excellent biocompatibility, dimensional stability under prolonged compression, and reduced risk of impingement on the surrounding tissue.

Similarly, La Rosa et al. [104] created an artificial IVD with a core made of hydrogel, an outer containment belt consisting of high-density polyethylene, and upper and lower containment plates made of the same plastic material of greater stiffness. The used materials were highly biocompatible and were able to withstand large deformations in elastic conditions when subjected to forward and back bending, lateral bending, torsion, and combined loading.

Zhu et al. [105] also prepared a biomimetic IVD composite scaffold that simulates the AF-NP structure. The researchers used oriented porous poly(l-lactide)/octa-armed polyhedral oligomeric silsesquioxanes fiber bundles for preparing the AF-like structure, whereas a gellan gum/poly (ethylene glycol) diacrylate double network hydrogel loaded with bone marrow MSCs simulated the NP structure. The construct showed similar mechanical properties to natural IVDs, providing good mechanical support for tissue repair and regeneration. Moreover, animal experiments revealed that the designed scaffold could maintain the disc space and produce new ECM.

**Figure 4:**
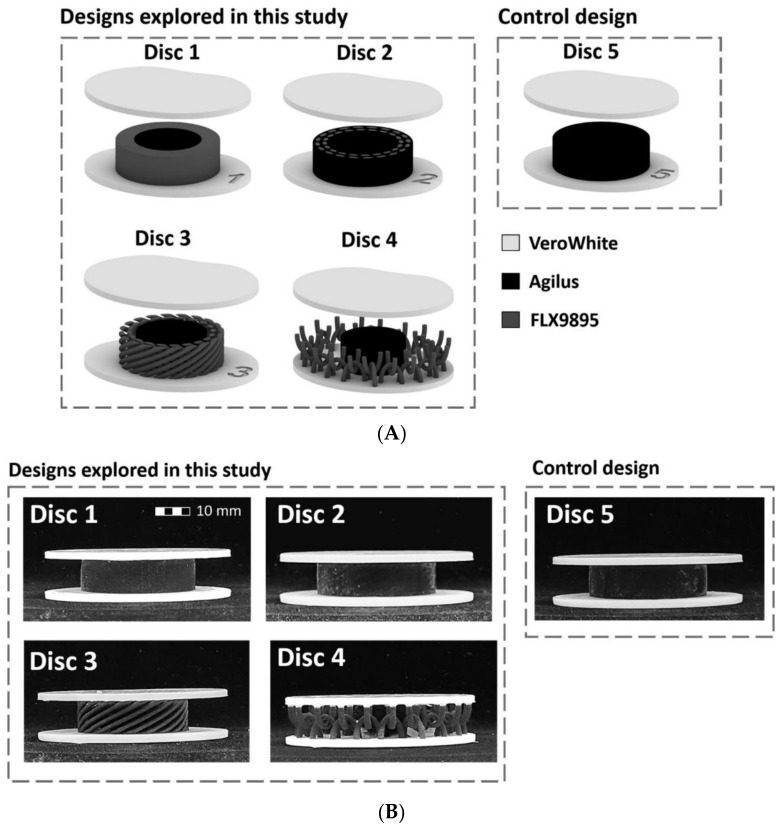
(**A**) Schematic representations and (**B**) 3D-printed specimens of the artificial IVDs designed by Yu et al. Reprinted from an open access source [106].

On a different note, Yu et al. [106] fabricated four types of biomimetic multi-material artificial spinal disc designs (Figure 4). The overall design of the artificial IVDs is similar, consisting of two stiff endplates and a compliant core. All the endplates were the same, made of the stiffest building material, referred to as VeroWhite (E ≈ 2 GPa), while the soft core is varied among the designs. Disc 1 presents a core with a softer central cylinder and an outer stiffer ring to mimic the functional stiffness gradient of native IVDs. Discs 2 and 3 have a criss-cross, fiber-like structure that simulates the fiber network in an IVD. Nonetheless, these two discs differ as follows: Disc 2 has a core analogous to a fiber-reinforced matrix composite, while Disc 3 presents a soft, central cylinder and a surrounding stiffer, criss-cross, fiber-like structure. Disc 4 is different, consisting of a soft, central cylinder and a chainmail-like structure that surrounds it and exhibits similar characteristics to the fiber network in the native IVD. Disc 5 was used as the control design, having a core made from a single material. The core materials are flexible, rubber-like, and are referred to as Agilus (E ≈ 0.5 MPa) and FLX9895 (E ≈ 5 MPa). All the four designed artificial spinal discs exhibited a viscoelastic behavior with nature-mimicking mobile instant helical axis and instant center of rotation, while Disc 4 also presented a distinct nature-mimicking nonlinear rotational response. Moreover, Discs 2–4 better mimicked the IVD’s structure than Disc 1, which only mimicked the IVD’s stiffness gradient. Thus, future studies can be performed for material optimization of Discs 2–4 to further enhance mimicking performances and create better IDD management options.

Therefore, creating suitable artificial IVDs is not only a matter of choosing the right materials but also requires investigations of different designs towards optimizing mechanical properties and biomimicry of the implants.

### 3.4. Other Emerging Strategies in Managing Intervertebral Disc Degeneration

#### 3.4.1. Exosomes

**Figure 5:**
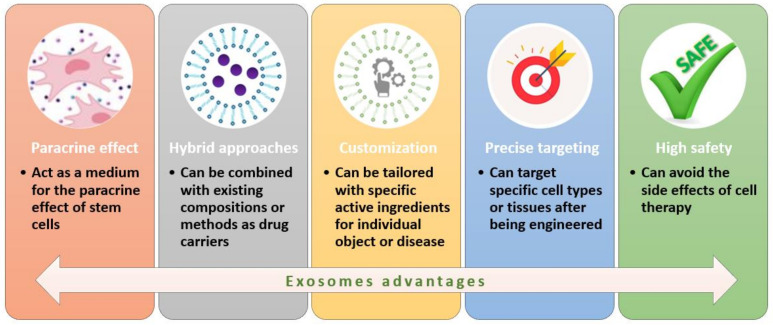
Advantages of using exosomes in regeneration therapies. Created based on information from [9].

The progress of tissue engineering and regenerative medicine has also enabled a better assessment of exosomes’ roles and potential in treating various diseases. These extracellular vesicles have a diameter of 30–150 nm, originate from intracellular multivesicular bodies (MVBs), and are released by almost all kinds of cells, including MSCs. In comparison to MSCs, MSC-derived exosomes present a series of advantages in promoting tissue regeneration [9,107,108,109] (Figure 5).

Tackling these advantages, Xia et al. [110] investigated the activity of MSC-derived exosomes for creating IDD therapeutics. The authors reported that exosomes exhibit anti-inflammatory properties against pathological NP cells via suppressing inflammatory mediators and NLRP3 inflammasome activation. In addition, exosomes might supply mitochondrial proteins to NP cells, restoring proper mitochondrial activity. In animal models, exosome treatment was shown to prevent degeneration progression. A similar study was conducted by Zhang and colleagues [111], who also observed that MSC-derived exosomes have an anti-pyroptosis role by suppressing the NLRP3 pathway, attributing this effect to miR-410 that could directly bind to NLRP3mRNA. Alternatively, Zhu et al. [112] reported that MSC-derived exosomes ameliorate NP cell apoptosis through a different mechanism. Specifically, the authors indicated that these exosomes reduce IL-1β-induced inflammatory cytokine secretion and MAPK signaling activation by targeting mixed-lineage protein kinase 3 (MLK3).

On a different note, Luo et al. [113] recently developed a therapeutic strategy for inhibiting IDD by the use of sphingosine kinase 2 (Sphk2)-engineered exosomes. In more detail, the researchers injected hydrogels modified with ECM of costal cartilage loaded with cartilage endplate stem cells (CESCs) overexpressing Sphk2. These cells produced the desired exosomes in situ, which further penetrated the annulus fibrosus and transported Sphk2 into the NP cells, where Sphk2 activated the (PI3K)/p-AKT pathway and the intracellular autophagy of NP cells, eventually resulting in the amelioration of IDD.

#### 3.4.2. RNA Approaches

Interesting therapeutic possibilities could also arise from the recently explored RNA-based strategies. For instance, Zhang et al. [114] exploited the potential of microRNAs in post-transcriptional gene modulation, investigating the role of microRNA-15a-5p (miR-15a-5p)/Sox9/NF-κB axis in relation to inflammation and apoptosis of murine NP cells. This study revealed that the downregulation of miR-15a-5p could increase Sox9 levels to activate p-p65 expression, inhibit NP cell apoptosis and inflammatory response through the NF-κB pathway, and stimulate NP cell proliferation in IDD animal models.

**Figure 6:**
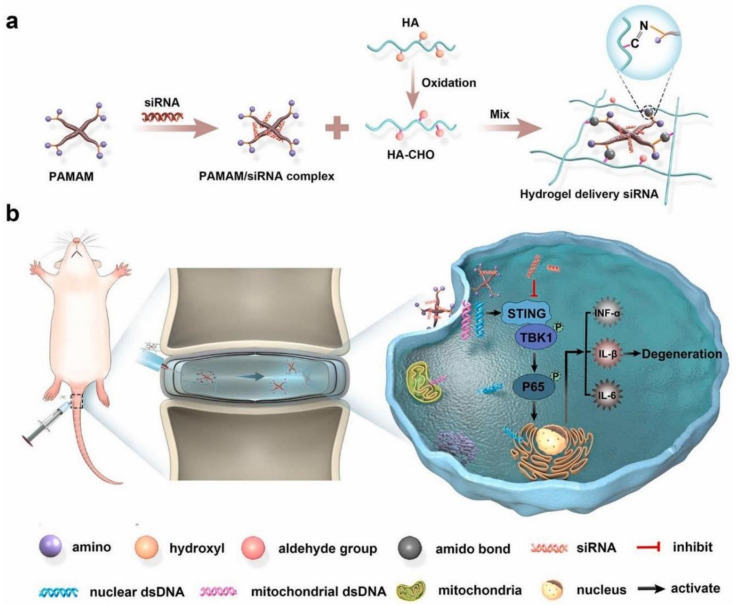
Schematic representation of the delivery system created by Chen et al. (**a**) Formation mechanism of the self-healing hydrogel with siRNA releasing property. (**b**) Mechanism of action against IDD. Reprinted from an open access source [116].

Given the involvement of DNA damage induced by ROS in the degeneration of NP cells, scientific interest has been drawn to monitoring this damage. In this respect, Guo et al. [115] explored the role of STING, the main effector of the cyclic GMP–AMP stimulator of interferon genes (cGAS-STING) signaling pathway that acts as a DNA-sensing mechanism. The authors observed that overexpression of STING promoted ECM degradation, apoptosis, and senescence of TBHP-treated and untreated NP cells, while a STING knock-down significantly reversed these effects, alleviating the development of puncture-induced IDD. With these aspects in mind, Chen and colleagues [116] recently proposed the use of a siSTING delivery hydrogel of aldehyde hyaluronic acid and poly(amidoamine)/siRNA complex for IDD treatment. This injectable and self-healing hydrogel was reported to efficiently and steadily silence STING expression in NP cells, easing inflammation and slowing IDD (Figure 6).

Another study revolving around siRNA was conducted by Banala et al. [117]. The researchers hypothesized that silencing Caspase 3 (which is involved in apoptosis) and *ADAMTS5* (which is involved in ECM degradation) can prevent the progression of IDD or even reverse it. A synergistic effect was reported in animal models receiving Cas3-AT5 siRNA formulation, with the treated intervertebral discs showing signs of recovery and regeneration 4 and 8 weeks after injection.

Differently, Hu et al. [118] recently investigated a new circRNA (circ_0022382) from human endplate chondrocytes. The authors discovered that circ_0022382 promoted the morphology of endplate chondrocytes by sponge-binding miR-4726-5p downregulation of target gene TGF-β3 expression, being able to alleviate IDD. These findings were also supported by in-vivo evidence, as the injection of circ_0022382 in a rat model was noticed to relieve IDD progression.

#### 3.4.3. Platelet-Rich Plasma

PRP represents the processed liquid fraction of autologous peripheral blood with a platelet concentration above the baseline, which has recently emerged as a promising therapeutic strategy for managing IDD [119]. Its treatment potential lies in PRP’s ability to decrease apoptosis and increase autophagy [120]. Other advantageous effects of PRP proved by in-vitro and in-vivo experiments count nutrient acquisition of seed cells, stimulation of cell proliferation and ECM regeneration, and restoration of IVD height [121].

Moreover, a literature survey of existing clinical studies in the field conducted by Muthu et al. [122] recently revealed that intradiscal PRP injection is beneficial in controlling the LBP associated with disc degeneration, despite not showing structural or functional improvements.

Nonetheless, an ongoing clinical trial (NCT04816747) that aims to investigate the precise effects of intradiscal and intra-articular injection of PRP in patients with early-stage lumbar IDD and facet joint syndrome might soon shed some light on the topic.

#### 3.4.4. Artificial Intelligence

Artificial Intelligence (AI) has increasingly become popular in the technological world, bringing promising progress to the medical system. AI has the potential to improve the prevention, screening, and treatment of diseases and the prediction of their prognosis, assisting healthcare professionals in making personalized decisions for each patient [123,124,125,126,127,128]. Even though the use of AI and machine learning (ML) techniques has not been widely investigated or adopted to diagnose spinal pain [129], a couple of studies have been recently published in the field.

Bradley and Rajendran [130] developed an ML-based tool for predicting the risk of IDD to aid in the earlier detection of this disease. Through the use of several ML algorithms (i.e., logistic regression, decision tree, artificial neural network, boosting, bagging, and random forest), application of the Synthetic Minority Oversampling Technique (SMOTE) method, and utilization of the cluster-then-predict technique, the developed tool could help identify disc degeneration, even in the early stages. Thus, this method could be helpful in closely monitoring and assessing the risk of disc degeneration.

Another ML-based approach is offered by Amol Soin et al. [129], who fed a decision tree ML software program with data collected from 246 patients with spinal pain. Patients had to complete an online form with 85 specific data points, such as demographic information, type of pain, pain score, pain location, pain duration, and functional status scores. The software managed to predict diagnoses with an accuracy rate of 72%, indicating the promising potential of AI in this clinical setting.

## 4. Conclusions

To summarize, IDD is a significant health burden whose current treatment faces limitations in terms of risks and long-term efficiency. Thus, the unmet need for new treatment options gained interdisciplinary scientific interest for deeper investigation into IDD’s causes and mechanisms towards envisaging more performant therapeutic options. Consequently, recent research focused on finding compounds that would act on the underlying factors of IDD rather than alleviating its symptomatology. In this respect, future promising formulations for IDD treatment might include melatonin, estrogen, naringin, icariin, resveratrol, quercetin, berberine, metformin, Vitamin D, or various growth factors. Moreover, cell-based therapies hold great promise for IVD regeneration, being able to restore damaged living tissues or at least slow down degenerative processes. Another highly investigated possibility is the development of artificial IVDs for replacing degenerate native discs. This option has recently explored the use of biocompatible and/or biodegradable materials to avoid the risk of long-term complications associated with the first generations of such implants.

In conclusion, various opportunities may come from further optimizing the above-discussed therapeutic strategies and translating them from preclinical testing to clinical trials. Moreover, currently completed clinical studies can be expected to bring advances in treating IDD and improve the quality of life of the millions of affected people in the near future.

## Figures and Tables

**Table 1:** Summary of key compounds for intervertebral disc degeneration (IDD) treatment.

Compound	Relevant Effects for IDD	Proposed Mechanism(s)
Melatonin	Antioxidant properties Anti-inflammatory activity Cell arrest inhibition Aggrecan and collagen II upregulation Collagen X downregulation Vascular invasion inhibition Calcification mitigation Damage repair acceleration	Activation of ERK1/2 signaling pathway Inhibition of NF-κB pathway
Estrogen	Autophagy stimulant Antioxidant properties Anti-inflammatory activity Apoptosis inhibition Catabolism reduction Anabolism upregulation	Activation of PI3K/Akt pathway Inhibition of NF-κB pathway
Naringin	Autophagy stimulant Antioxidant properties Anti-inflammatory activity Apoptosis attenuation Upregulation of aggrecan, BMP-2, and Sox6 Downregulation of TNF-α and MMP3	Activation of PI3K/Akt pathway
Icariin	Antioxidant properties Anti-inflammatory activity ECM preservation Stem cells recruitment Upregulation of chemotactic cytokines Downregulation of Caspase-3 and Bax	Inhibition of MAPK pathway Inhibition of NF-κB pathway
Resveratrol	Autophagy stimulant Antioxidant properties Anti-inflammatory activity Apoptosis attenuation Aggrecan and collagen II upregulation GAG production stimulation	Activation of PI3K/Akt pathway
Quercetin	Autophagy stimulant Antioxidant properties Anti-inflammatory activity Apoptosis inhibition ECM degradation prevention	Inhibition of p38 MAPK signaling pathway Inhibition of NF-κB pathway
Berberine	Autophagy stimulant Antioxidant properties Anti-inflammatory activity Apoptosis prevention ER stress modulation Inhibition of matrix-degrading enzymes production Upregulation of ECM-catabolic factors Downregulation of ECM-anabolic factors	Inhibition of NF-κB pathway
Metformin	Autophagy stimulant Anti-inflammatory activity Apoptosis attenuation Cellular senescence inhibition Reduction of hypermethylation level of SOX9 promoter Upregulation of anabolic genes Downregulation of catabolic genes	Inhibition of NF-κB pathway Blockage of HMGB1 translocation
Vitamin D	Antioxidant properties Anti-inflammatory activity Apoptosis inhibition Cellular senescence delay Aggrecan and collagen II upregulation Collagen X downregulation	Inhibition of NF-κB pathway

**Table 2:** Clinical trials involving growth factor therapy for treating intervertebral disc degeneration. The studies were retrieved from clinicaltrials.gov with search keywords “Condition or disease = Intervertebral Disc Degeneration” and “Other terms = growth factor”; the relevant search results were manually selected.

ClinicalTrials.Gov Identifier	Official Title	Intervention/ Treatment	Enrollment	Intervention Model	Phase	Status (as Reported until 10 May 2022)
NCT01158924	A Phase I/IIa, Multicenter, Open-label, Clinical Trial to Evaluate the Safety, Tolerability and Preliminary Effectiveness of Single Administration Intradiscal rhGDF-5 for the Treatment of Early Stage Lumbar Disc Degeneration	Drug: Intradiscal rhGDF-5	40 participants	Single Group Assignment	Phase 1 Phase 2	Completed
NCT00813813	Phase I/II, Multicenter, Open-label, Single Administration, Dose Finding, Clinical Trial to Evaluate the Safety and Tolerability of Intradiscal rhGDF-5 for the Treatment of Early Stage Lumbar Disc Degeneration	Drug: Intradiscal rhGDF-5	32 participants	Single Group Assignment	Phase 1 Phase 2	Completed
NCT01182337	A Multicenter, Randomized, Double-blind, Placebo Controlled, Clinical Trial to Evaluate the Safety, Tolerability and Preliminary Effectiveness of Single Administration Intradiscal rhGDF-5 for the Treatment of Early Stage Lumbar Disc Degeneration	Drug: Intradiscal rhGDF-5 Drug: Vehicle control	31 participants	Parallel Assignment	Phase 1 Phase 2	Completed
NCT01124006	A Multicenter, Randomized, Double-blind, Placebo Controlled, Clinical Trial to Evaluate the Safety, Tolerability and Preliminary Effectiveness of 2 Doses of Intradiscal rhGDF-5 (Single Administration) for the Treatment of Early Stage Lumbar Disc Degeneration	Drug: Intradiscal rhGDF-5 Other: Water for injection	24 participants	Parallel Assignment	Phase 2	Completed
NCT04816747	Intradiscal and Intra-articular Injection of Autologous Platelet-Rich-Plasma (PRP) in Patients With Lumbar Degenerative Disc Disease and Facet Joint Syndrome: A Prospective, Single-arm, Open Label Clinical Trial	Biological: Autologous PRP	50 participants (estimated)	Single Group Assignment	Phase 3	Not yet recruiting

## Data Availability

Not applicable.

## Abbreviations

AF	annulus fibrous
AI	artificial intelligence
AMPK	AMP-activated protein kinase
Bax	BCL-2-associated X protein
BC	bacterial cellulose
BCL-2	B-cell lymphoma 2
BMAC	bone marrow aspirate concentrate
BMP	bone morphogenetic protein
CCL-5	C-C motif chemokine ligand 5
CEP	cartilaginous endplates
cGAS	cyclic GMP–AMP
E2	17β-estradiol
ECM	extracellular matrix
ERK	extracellular signal-regulated kinase
ESC	embryonic stem cells
GAG	glycosaminoglycan
GDF-5	growth differentiation factor 5
GMP	good manufacturing practice
IDD	intervertebral disc degeneration
IFN-γ	interferon-gamma
IGF-1	insulin-like growth factor 1
IL	interleukin
iPSC	induced pluripotent stem cells
IVD	intervertebral disc
LBP	low back pain
LC3	light chain 3
MAPK	mitogen-activated protein kinase
ML	machine learning
MLK3	mixed-lineage protein kinase 3
MMP	matrix metalloproteinase
MSC	mesenchymal stem cells
mtROS	mitochondrial ROS
MVB	multivesicular bodies
NF-κB	nuclear factor kappa B
NLRP3	NLR family pyrin domain containing 3
NP	nucleus pulpous
Nrf-2	nuclear factor erythroid 2–related factor 2
p-Akt	phosphorylated Akt
PCL	poly(caprolactone)
PDGF	platelet-derived growth factor
P-GSK-3β	phospho-glycogen synthase kinase-3 beta
PHEMA	poly(2-hydroxyethylmethacrylate)
PI3K/Akt	phosphatidylinositol-3-kinase/protein kinase B
PLA	polylactic acid
P-mTOR	phospho-mammalian target of rapamycin
PRP	platelet-rich-plasma
PVA	polyvinyl alcohol
RANKL	receptor activators for nuclear factor-κB ligand
REC	rapidly expanding clone
rhGDF-5	recombinant human GDF-5
ROS	reactive oxygen species
SCN	stem cell niche
SDF-1	stromal-cell-derived factor-1
SERM	selective estrogen receptor modulators
SIRT1	sirtuin 1
Sox	SRY-Box Transcription Factor
Sphk2	sphingosine kinase 2
STING	stimulator of interferon genes
TDR	total disc replacement
TGF-β	transforming growth factor beta
TNF-α	tumor necrosis factor-alpha
TRAP	tartrate-resistant acid phosphatase
